# Beyond the Bronchi: Uncovering a Rare Mid-esophageal Dieulafoy Lesion in Non-massive Hemoptysis

**DOI:** 10.7759/cureus.91654

**Published:** 2025-09-05

**Authors:** Kevin T Huynh, Nina Kanase, Timothy N Holbrook, Lavanya Srinivasan

**Affiliations:** 1 Internal Medicine, Baylor Scott & White All Saints Medical Center, Fort Worth, USA; 2 Pulmonology and Critical Care, Baylor Scott & White All Saints Medical Center, Fort Worth, USA

**Keywords:** dieulafoy lesion, flexible bronchoscopy, hematemesis, hemoptysis of endobronchial and parenchymatous origin, nasopharyngolaryngoscopy, non-massive hemoptysis, pseudohemoptysis, pulmonary hemorrhage, true hemoptysis, upper endoscopy

## Abstract

Hemoptysis has an annual incidence in the United States (US) of 0.1-0.2% in the outpatient and inpatient clinical settings, respectively. True hemoptysis is described in medical literature as the expectoration of blood-tinged sputum originating from the lower respiratory tract. The severity of hemoptysis ranges from non-massive to massive. By contrast, pseudohemoptysis is the expectoration of blood-tinged sputum originating from the upper gastrointestinal (GI) or nasopharyngeal tract. We report the case of a 91-year-old woman undergoing stepwise evaluation of non-massive, recurrent hemoptysis of three-month duration with chest pain. Extensive radiological imaging, including computed tomography angiography (CTA) of the thoracic, head, and neck, did not reveal active extravasation of vascular vessels. Fiberoptic bronchoscopy with bronchoalveolar lavage (BAL) demonstrated mild erythema of the right upper lobe (RUL) without an obvious source of bleeding. Broader workup of hemoptysis with gastroenterology consultation for endoscopic ultrasound (EUS) and esophagogastroduodenoscopy (EGD) revealed a rare mid-esophageal Dieulafoy lesion with stigmata of recent bleeding. The defect is then repaired with improvement in clinical symptoms of hemoptysis. This case highlights a rare instance of an esophageal Dieulafoy lesion masquerading as hemoptysis.

## Introduction

Hemoptysis occurs in approximately 0.1% of outpatients and 0.2% of inpatients in clinical settings in the United States [1]. Initial evaluation of hemoptysis includes a pertinent medical history, focused physical exam, and hemodynamic assessment to assess the severity [2]. Hemoptysis ranges from non-massive to massive hemoptysis, with the former often being self-limiting. Further investigation of etiology typically involves two-view chest radiography, contrast-enhanced computed tomography (CT), computed tomography angiography (CTA) of the chest, or multidetector computed tomography (MDCT) [3,4].

By contrast, massive hemoptysis is associated with a higher risk of interval clinical decompensation. The degree of copious blood-tinged sputum production places the patient at higher risk for asphyxiation and exsanguination in the context of acute blood loss anemia. Massive hemoptysis is potentially life-threatening. Available evidence indicates the initial diagnostic evaluation for massive hemoptysis also includes the aforementioned radiographic imaging for non-massive hemoptysis [3]. Although expert consensus continues to debate the definitive range for life-threatening massive hemoptysis, it is generally accepted to be >50 milliliters (mL) of blood volume in a single expectoration event, as little as 100-200 mL over 24 hours or >1000 mL over 48 hours [1,3,5]. At any rate, other sources debate that respiratory and cardiovascular hemodynamics are more significant than the precise categorization of massive hemoptysis [6]. The presence of blood volume loss can significantly impair gas exchange at the level of the alveoli, leading to dramatic shifts in acid and base imbalance and worsening overall hypoxemia [6]. 

The combination of acute blood loss anemia, increasing risks of asphyxiation, and hemodynamic instabilities that are exacerbated by underlying comorbidities inevitably leads to respiratory failure [6]. Immediate intubation with subsequent rigid bronchoscopy to isolate bleeding bronchial segments becomes the mainstay of management [1,7-12]. Pulmonology consultation for diagnostic bronchoscopy to determine hemoptysis of endobronchial or parenchymatous origin is warranted when radiological imaging and etiology of hemoptysis are inconclusive. A broader work-up must be initiated to investigate other causes of cryptogenic hemoptysis related to pseudohemoptysis and hematemesis [6].

On occasion, hemoptysis presents with hematemesis that originates from the gastrointestinal (GI) or nasopharyngeal tract, known as pseudohemoptysis. Esophageal Dieulafoy lesions represent an extremely rare etiology of pseudohemoptysis, which can be visualized through upper endoscopic evaluation. Dieulafoy lesions are described in the current literature as a dysplastic, abnormally dilated submucosal artery along any portion of the GI tract and, on occasion, in the bronchopulmonary system [13]. Dieulafoy lesions are rare and recognized as unexplained causes of recurrent hematemesis [13]. The aberrant anatomy results in incomplete tapering of arteriolar walls near the submucosal surface. From literature reviews, subtle mucosal protrusions and visible pulsations are often described [14]. Detection in endoscopic interventions is often elusive without pathognomonic inflammatory or ulcerative features; however, minor trauma and rupture can lead to clinical manifestations of hematemesis, melena, hematochezia, longstanding acute blood loss anemia, and, less commonly, recurrent hemoptysis [13-14].

## Case presentation

We present a 91-year-old female who presented with recurring hemoptysis described as bright, red blood with intermittent blood-tinged sputum and clots that had been ongoing over the last three months. She reported severe non-radiating midsternal epigastric pain and palpitations aggravated by coughing spells and physical exertion. The midsternal chest pain was described as a stabbing sensation without radiation, and the chest pain had previously always been relieved by TUMS tablets. She denied large-volume emesis. She had a pertinent past medical history of paroxysmal atrial fibrillation, a known hiatal hernia complicated by gastroesophageal reflux disease (GERD), coronary artery disease (CAD), and hypothyroidism. Social history was positive for a 20-pack-year smoking history and recent alcohol cessation three years ago. Family history was significant for ovarian cancer in her mother, but she did not have a personal history of malignancy.

**Figure 1:**
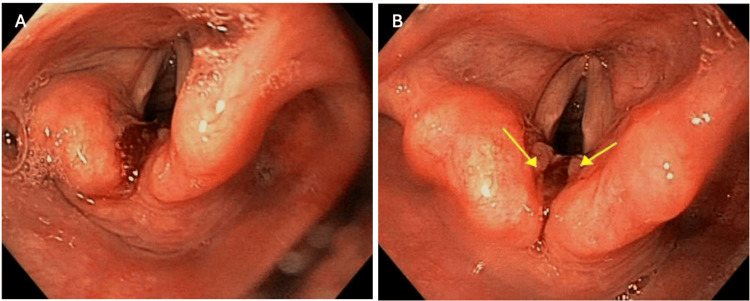
A) Esophagogastroduodenoscopy (EGD) blood at the site of the arytenoid without active oozing. B) Arrows indicating possible tear in arytenoids. This lesion was thought to be initialy etiology of recurring production of bloody mucus which prompted further consultation with ENT.

Of note, she was hospitalized three months ago for a similar presentation of hemoptysis and chest pain. She received extensive radiological imaging per the recommended literature guideline, and a broad work-up was conducted to investigate the etiology of her hemoptysis. CTA chest was negative for pulmonary embolism. Evidence of faint bilateral multilobar ground-glass opacities with subsegmental and mild consolidative opacity atelectasis was seen in the upper right lobe. There was no obvious bronchial vessel lesion that would explain bloody-tinged mucus. CTA neck showed mild bilateral proximal internal carotid artery (ICA) stenosis, severe narrowing of the left vertebral artery, and C6-C7 moderate spinal cord narrowing. A tear in the arytenoids without active oozing was found during the esophagogastroduodenoscopy (EGD), as visualized in Figure 1, and the remaining esophagus was confirmed as normal. On nasopharyngolaryngoscopy, there was dried blood in the upper airway. However, the nasal cavity was without polyposis or purulence. The nasopharynx, base of tongue, vallecula, and larynx were without mass lesions or pooling of secretions. There was no visible tear noted to the arytenoids or active bleeding from a nasopharyngeal origin. Active phonation was attempted with normal vocal cord movement. Next, a flexible bronchoscopy with bronchial washing and lavage of bilateral lungs was performed. The right upper lobe was noted to have mild erythema, but there was no obvious source of bleeding. Clinical symptoms improved, and the patient was discharged from the hospital without a clear etiology of non-massive hemoptysis.

**Figure 2:**
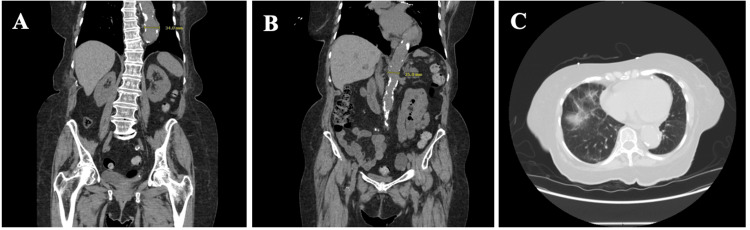
A) CTAP without contrast, coronal ectasia of descending aorta measuring 3.4 cm. B) Coronal ectasia of AAA measuring 2.5 cm. C) Axial view, mild patchy ground-glass density in bilateral lung zones.

Three months later, the patient was re-admitted due to expectoration of blood-streaked mucus and midsternal discomfort, raising concern for recurrent chronic hemoptysis. Oxygen saturation and vitals remained stable, as shown in Table 1, and the patient was without signs and symptoms of acute hypoxic respiratory failure. Initial laboratory presentation during this admission is presented in Table 2 and Table 3. Given the slight normocytic anemia and location of hematemesis, a clinical judgment was made not to proceed with the fecal occult blood test (FOBT). An overall downtrend in hemoglobin from 14.0 g/dL three months ago to 11.0 g/dL was detected. The iron and total iron binding capacity (TIBC) panel were consistent with iron deficiency anemia secondary to hemoptysis, as shown in Table 4. CT abdomen pelvis without contrast did not reveal an acute bleed; however, there were incidental findings of ectasia of the descending thoracic aorta, abdominal aortic aneurysm (AAA), and patchy ground glass density in bilateral lung zones, as shown in Figure 2.

**Table 1:** Vital signs on admission and six hours after admission

Component	Reference range and units	Value on admission	Value six hours since admission
Temperature	97–99 (°F)	97.6	97.7
Pulse	60–100 BPM	73	66
Respiratory rate	12–20 RR	18	18
Blood pressure	Systolic = 120 mmHg; diastolic =80 mmHg	147/70 mmHg	119/58 mmHg
Pulse oximeter	90–100%	96% on room air	100% on room air

**Table 2:** Complete blood count

Component	Reference range and units	Value	Classification
White blood cells (WBC)	4.1–10.5 K/uL	6.6	Normal
Red blood cells (RBC)	3.99–5.46 M/uL	3.56	Low
Hemoglobin (Hgb)	12.2–15.3g/dL	11	Low
Hematocrit (HCT)	36.4–46.8 %	34.4	Low
Mean corpuscular volume (MCV)	82–99 fL	96.6	Normal
Mean corpuscular hemoglobin (MCH)	26.6–33.2 pg	30.9	Normal
Mean corpuscular hemoglobin concentration (MCHC)	31.1–35.2 g/dL	32	Normal
Platelet (PLT)	160–397 K/uL	226	Normal

**Table 3:** Comprehensive metabolic panel

Component	Reference range and units	Value	Classification
Glucose	70–99 mg/dL	93	Normal
Sodium level	136–145 mmol/L	140	Normal
Potassium level	3.4–4.5 mmol/L	3.9	Normal
Chloride	98–107 mmol/L	107	Normal
Anion gap	4–14 mmol/L	3	Low
Carbon dioxide	22–29 mmol/L	27	Normal
Blood urea nitrogen (BUN)	7–18 mg/dL	18	Normal
Creatinine	0.55–1.02 mg/dL	0.84	Normal
BUN/creatinine ratio	7–25	21	Normal
Estimated glomerular filtration rate (eGFR)	>=60 mL/min/1.73m^2^	58	Low
Calcium	8.5–10.1 mg/dL	8.8	Normal
Protein, total	6.4–8.2 g/dL	5.6	Low
Albumin	3.4–5.0 g/dL	3	Low
Bilirubin, total	1.7–3.3 g/dL	0.3	Normal
Alkaline phosphatase	45–117 U/L	70	Normal
Aspartate aminotransferase (AST)	15–37 U/L	15	Normal
Alanine aminotransferase (ALT)	13–56 U/L	19	Normal

**Table 4:** Iron and TIBC Panel

Iron and TIBC Panel		
Component	Reference Range and Units	Value
Iron	50 – 70 ug/dL	39
Iron Binding Capacity, Total	250 – 450 ug/dL	244
Transferrin saturation	15 – 50%	16
Ferritin	16 – 288 ng/mL	66

Pulmonology and gastroenterology were consulted to investigate the source of hemoptysis and hematemesis, with repeat flexible bronchoscopy and EGD performed in tandem. Bronchoscopy demonstrated mild inflammation in the right lower lobe bronchi, and the remaining right segmental and subsegmental bronchi appeared normal. The left main stem segmental and subsegmental bronchi appeared normal as well. There was no evidence of bleeding in the posterior pharyngeal airway or around the vocal cords. Upper EGD revealed a normal upper and lower third of the esophagus. However, a vascular, non-bleeding lesion with a cherry-red tinge in the middle third of the esophagus was hidden within the esophageal fold with clinical suspicion of a Dieulafoy lesion. There was clear evidence of mucosal disruption that started to ooze at the proximal end. Biopsy of the Dieulafoy lesion was not attempted due to suspicion of underlying vasculature. There was no blood upon further inspection of the stomach and duodenum.

**Figure 3:**
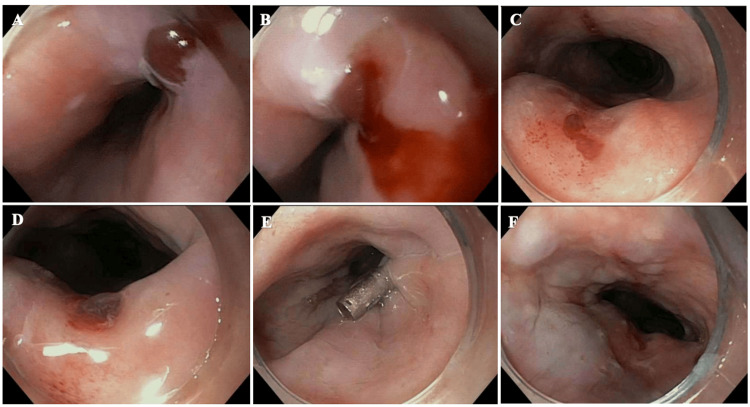
Esophagogastroduodenoscopy with demonstration of the mid-esophageal ulcer and hemostatic clip placement A) Mid-esophageal ulcer with cherry-tinge. B) Esophageal ulcer with oozing. C) Mid-esophageal ulcer redemonstrated.  D) Underlying vasculature suspicious for Dieulafoy lesion. E) Placement of hemostatic clip at the proximal tissue edges. F) Submucosal wall thickening adjacent to the vascular lesion.

Endoscopic ultrasound (EUS) and esophagogastroduodenoscopy (EGD) were attempted and confirmed the mid-esophageal lesion, approximately at 32 cm from the incisors, characterized by an ulcer actively oozing blood (Figure 3). The defect was repaired by approximation of the tissue edges and placement of one hemostatic clip. Endosonographically, there was wall thickening visualized at the area adjacent to the esophageal lesion, primarily due to thickening of the submucosa.

In the event of refractory bleeding, vascular surgery and interventional radiology were consulted to provide additional management options. However, after EGD intervention of the Dieulafoy lesion, the patient maintained clinical stability and was discharged to close follow-up with pulmonology. Three weeks later, the patient was still without recurrence of hemoptysis or hematemesis. 

## Discussion

Hemoptysis may represent an underlying infectious or inflammatory processes of the airways that commonly present as pneumonia, acute and chronic bronchitis, or tuberculosis [1-2,14-15]. Moreover, underlying lung malignancies also contribute to the burden of hemoptysis [1-2,14-15]. Pseudohemoptysis from the upper GI tract or nasopharyngeal airways is important to consider when common hemoptysis etiologies are ruled out.

The presentation of recurrent hemoptysis with blood-streaked sputum warrants a comprehensive investigation to differentiate between true hemoptysis and pseudohemoptysis. A recognition of non-massive and massive hemoptysis determines the urgency of evaluation, as hemodynamic instability exponentially increases the risk of acute hypoxic respiratory failure from asphyxiation or exsanguination from hemorrhage. Urgent flexible bronchoscopy is recommended as a first-line intervention in hemodynamically unstable (tachycardia, hypotension) patients with life-threatening massive hemoptysis, according to recent studies [1,5]. Early flexible bronchoscopy can be both diagnostic and therapeutic: it localizes the bleeding site, achieves immediate hemostasis via endobronchial tamponade, and prevents blood translocation into proximal bronchial segments [14-15]. If severe pulmonary hemorrhage continues, an early conversion to rigid bronchoscopy is warranted to provide sufficient oxygenation of the non-bleeding lung through unilateral intubation [15]. Moreover, MDCT imaging is adequately sensitive to identify hemorrhage of endobronchial and parenchymal origin with accurate mapping of bronchial vasculature. Subsequent endovascular bronchial artery embolization is then recommended as the first-line treatment, achieving 75-98% hemostasis [5,15]. 

The dual circuit consisting of pulmonary and bronchial arteries supplies the pulmonary system, yielding approximately 99% and 1% of cardiac output, respectively [10-11]. Bronchial arteries commonly branch off from the descending thoracic aorta at the T5-T6 vertebral level. While the bronchial vasculature receives approximately 1% of cardiac output to supply the bronchial circulation, pressure gradients are higher in comparison to its counterpart. Consequently, many cases of severe pulmonary hemorrhage requiring early endobronchial interventions are attributed to approximately 90% from bronchial arteries, 5% from pulmonary arteries, and 5% from non-bronchial systemic arteries [1,8-9,14]. Invasive procedures such as transbronchial biopsy can ultimately exacerbate fatal hemorrhage due disruption of the underlying rich supply of arterial vascular network [9-10]. In our case, standard bronchoscopy and imaging failed to reveal a pulmonary source of hemoptysis. Through investigation of pseudohemoptysis causes, a rare Dieulafoy lesion in the mid-esophagus was visualized on EGD. Due to intermittent bleeding and subtle endoscopic appearances, Dieulafoy lesions can often be missed on initial endoscopy.

Endoscopic management of Dieulafoy lesions lacks formal consensus, with available evidence limited to small case series or retrospective reports [16]. Mechanical endoscopic therapies such as endoscopic band ligation or hemoclipping are the most effective, with success rates near 95% and recurrence under 10% [17]. Argon plasma coagulation is another effective endoscopic therapy, whereas epinephrine injection alone is inadequate. For cases refractory to endoscopic intervention, selective arterial embolization via angiography or surgical wedge resection is preferred. In our case, successful hemostasis was achieved with mechanical therapy.

## Conclusions

This case highlights a rare mid-esophageal Dieulafoy lesion presenting as a recurrent, non-massive hemoptysis. It underscores the intricacy of the stepwise, complex clinical investigation of hemoptysis. A gastroenterology consultation is warranted to rule out pseudo-hemoptysis with EGD evaluation. ENT consultation is warranted to further assess the vocal cords and structures with nasopharyngolaryngoscopy. In conclusion, we emphasize the importance of considering gastrointestinal and nasopharyngeal sources to rule out when pulmonary evaluations are inconclusive.
